# F224. UTILITY OF SALIVA FOR MONITORING OF CLOZAPINE LEVELS

**DOI:** 10.1093/schbul/sby017.755

**Published:** 2018-04-01

**Authors:** Jimmy Lee, Jie Yin Yee, Yuen Mei See, Charmaine Tang, Boon Tat Ng, Gary Remington, Balram Chowbay

**Affiliations:** 1 Institute of Mental Health, Singapore; 2 Centre for Addiction and Mental Health (CAMH), University of Toronto; 3 National Cancer Centre, Singapore

## Abstract

**Background:**

Clozapine has specific indication for use in Treatment Resistant Schizophrenia (TRS) with guidelines for therapeutic drug monitoring. Though a plasma level > 350 ng/ml has been cited as the therapeutic threshold, many other studies have shown poor or inconsistent associations between blood levels of clozapine and side effects. Clozapine is about 95% bound to plasma proteins with a small biologically active fraction. Saliva presents as a promising alternative for therapeutic drug monitoring where clozapine levels would be at equilibrium with free unbound clozapine in the plasma. This provides the added advantage of a potentially stronger relationship with efficacy and adverse effects when compared to plasma levels because of saliva’s closer representation of biologically-active clozapine. Salivary collection is also non-invasive and can be sampled serially for more precise evaluation of intra-individual variations. In the present investigation, we set out to evaluate the agreement and comparative clinical utility between plasma and salivary clozapine levels.

**Methods:**

53 participants with schizophrenia and on stable doses of clozapine for at least 2 weeks were recruited for the study. Participants had to undergo a clinical interview and the SCID, PANSS, plus side effect scales were administered. Symptomatic remission status was defined using the symptom criteria proposed by Andreasen et al (2005). A fasting sample of venous blood and salivary sample were collected at the same time. Assays for clozapine and norclozapine were performed using high performance liquid chromatography. A total of 106 saliva and plasma samples have been analysed.

**Results:**

Our results showed strong correlations between plasma and salivary levels of clozapine (r=0.61, P<0.05) and norclozapine (r=0.63, P<0.05). Twenty (37.7%) participants achieved symptomatic remission at the time of recruitment. Non-remitters had a significantly higher level of plasma clozapine. Thirty-one (93.9%) participants in the non-remitter group have plasma clozapine levels greater than 350ng/ml.

**Discussion:**

Our study shows potential for salivary samples to be an alternate non-invasive source for therapeutic drug monitoring of clozapine. This will be useful in serial monitoring of clozapine levels to evaluate treatment adherence and fluctuating pharmacokinetic profiles. There is a significant proportion of patients who do not achieve symptomatic remission on clozapine, which highlights a pressing need to identify new pharmacological agents or modalities of treatment.

